# Efficacy of a Newly Developed Guidewire for Selective Biliary Cannulation: A Multicenter Randomized Controlled Trial

**DOI:** 10.3390/jcm12103440

**Published:** 2023-05-12

**Authors:** Sung Yong Han, Sung Ill Jang, Dong Hee Koh, Jong Hyun Lee, Dong Uk Kim, Jae Hee Cho, Kyong Joo Lee, Seong-Hun Kim, Min Je Sung, Chang-Il Kwon

**Affiliations:** 1 Department of Internal Medicine, Pusan National University School of Medicine, Biomedical Research Institute, Pusan National University Hospital, Busan 49421, Republic of Korea; mirsaint@hanmail.net (S.Y.H.); keiasikr@nate.com (J.H.L.); amlm3@hanmail.net (D.U.K.); 2 Department of Internal Medicine, Gangnam Severance Hospital, Yonsei University College of Medicine, Seoul 03722, Republic of Korea; aerojsi88@gmail.com (S.I.J.); jhcho9328@gmail.com (J.H.C.); 3 Department of Internal Medicine, Hallym University Dongtan Sacred Heart Hospital, Hwaseong 18450, Republic of Korea; kyongjoolee1214@gmail.com; 4 Division of Gastroenterology, Department of Internal Medicine, Jeonbuk National University Medical School and Research Institute of Clinical Medicine of Jeonbuk National University-Biomedical Research Institute of Jeonbuk National University Hospital, Jeonju 54907, Republic of Korea; shkimgi@jbnu.ac.kr; 5 Digestive Disease Center, CHA Bundang Medical Center, CHA University School of Medicine, Seongnam 13496, Republic of Korea; mj1744@hanmail.net (M.J.S.); mdkwon@naver.com (C.-I.K.)

**Keywords:** biliary cannulation, guidewire, ERCP

## Abstract

Background and Aims: Various guidewires are used for biliary cannulation, and each one has its own characteristics affecting its effectiveness. This study aimed to measure the basic properties and evaluate the effectiveness of a newly developed 0.025-inch guidewire for selective biliary cannulation. Methods: A total of 190 patients at five referral hospitals were randomly allocated to undergo selective biliary cannulation using the newly developed guidewire (NGW group, *n* = 95) or a conventional guidewire (CGW group, *n* = 95). The primary outcome was the selective biliary cannulation rate in naïve papillae. The secondary outcome was to measure the NGW basic properties, compare them with those of the CGW, and analyze the importance of basic property differences. Results: There were no significant differences between the groups in the baseline characteristics. The primary outcome (75.8% vs. 84.2%, *p* = 0.102) and adverse event rate (6.3% vs. 4.2%, *p* = 0.374) were similar in both groups. However, compared with the CGW group, the NGW group showed a higher number of ampulla contacts (2.58 vs. 2.02, *p* = 0.011) and longer cannulation time (216.5 vs. 135.1 s, *p* = 0.016). Furthermore, the NGW group had higher maximum friction (34.6 ± 1.34 vs. 30.2 ± 4.09), lower stiffness, and better elastic resiliency. In the multivariate analysis, a curved-tip GW (OR = 0.26, 95% CI 0.11–0.62, *p* = 0.002) and normal papillary shape (OR = 0.39, 95% CI 0.17–0.86, *p* = 0.021) were contributing factors for successful selective biliary cannulation. Conclusions: The NGW group had high friction and low stiffness, characteristics affecting biliary cannulation. Clinically, the NGW group had similar success and adverse event rates as the CGW, but they showed a higher number of ampulla contacts and longer cannulation time.

## 1. Introduction

Endoscopic retrograde cholangiopancreatography (ERCP) is an important standard procedure for treating pancreaticobiliary diseases. A guidewire is an essential instrument for supporting and providing direction for the instrument in ERCP. In selective biliary cannulation via ERCP, the guidewire-assisted technique showed a higher primary biliary cannulation success rate and lower adverse event rate, including post-ERCP pancreatitis (PEP), than the contrast injection technique [1,2,3]. Moreover, the European Society of Gastrointestinal Endoscopy recommends the guide-wire-assisted technique for primary biliary cannulation [4].

The type of guidewire is selected according to the technical skills and convenience of the endoscopist. Recently, a high-performance 0.025-inch hydrophilic-coated guidewire with a nitinol core similar to 0.035 inch and a flexibility of 0.025 inch has been developed and widely used [5,6]. The 0.025-inch guidewire did not show a better performance status than the 0.035-inch guidewire in selective biliary cannulation [7,8]. However, a previous study showed that the 0.025-inch guidewire had a shorter cannulation time and less contact with the ampulla [6]. Furthermore, passing through a biliary stricture after cannulation and device exchange could be carried out more easily with the 0.025-inch guidewire than with the 0.035-inch guidewire [7,9].

Guidewire characteristics are determined by several factors, such as the flexibility and shape of the tip, the hydrophilic coating method, the rigidity of the shaft, and its visibility in fluoroscopy [9]. Even with the same diameter, guidewire performance may differ depending on the characteristics. Therefore, the basic properties of the newly developed 0.025-inch guidewire (NGW) should be measured using mechanical testing, and its performance should be compared with that of a conventional guidewire (CGW).

The shape of the guidewire tip is another important factor in selective biliary cannulation. Curved- or straight-tip guidewires are most commonly used in ERCP. Although new tip types have been studied, including the J and loop types, they did not have better success rates compared to the standard type [10,11]. Thus, it remains unclear which tip shape is most suitable for biliary cannulation.

Based on the above, the purpose of this study was to compare the effectiveness of the 0.025-inch NGW and CGW for primary selective biliary cannulation and to measure the basic properties of the NGW and compare them with those of the CGW to analyze the importance of basic property differences.

## 2. Materials and Methods

### 2.1. Clinical Study

#### 2.1.1. Study Design and Population

The study was conducted in accordance with the ethical guidelines of the Declaration of Helsinki (as revised in 2013). The study protocol was approved by the Institutional Review Board of our hospital (approval no. HDT 2021-08-014) and registered with no. KCT0006688 at CRIS (clinical research information service) at cris.nih.go.kr on 28 October 2021. All patients provided written informed consent for participation in the study.

**Figure 1:**
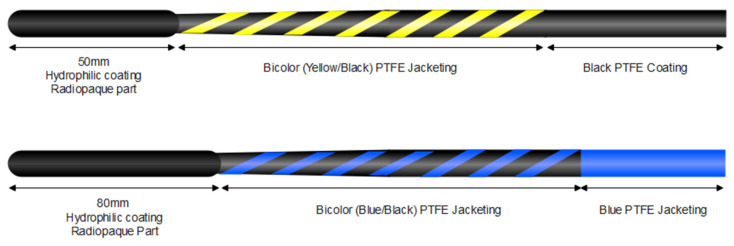
Schematic structure of each guidewire. Upper figure: 0.025-inch Jagwire^TM^ Revolution (Boston Scientific, USA). Lower figure: 0.025-inch Targetsure (Koswire, Republic of Korea).

The patients were competitively enrolled from five referral hospitals between January and June 2022. They were randomly allocated into the newly developed guidewire group (NGW; Targetsure, Koswire, Busan, Republic of Korea) or the conventional guidewire group (CGW; Jagwire^TM^ Revolution; Boston Scientific, Marlborough, MA, USA) (Figure 1). Randomization was performed by opening the randomization envelope after the patients agreed to participate in the study and signed the informed consent forms. We do not control the type of guidewire (straight or curved type), and the straight type and curved type were selected according to the endoscopist’s preference.

The inclusion criteria were as follows: (1) age ≥ 18 years and (2) first-in-life ERCP procedure for diagnosis and/or treatment (naïve papilla). The following were the exclusion criteria: (1) surgically altered anatomy (Billroth I or II, Roux-en-Y, and Whipple’s operation); (2) pancreatitis, including biliary pancreatitis; (3) ERCP performed for pancreatic disease, such as pancreatic duct stricture, stone, etc.; (4) difficulty in duodenoscope insertion due to duodenal stricture or gastric outlet obstruction; (5) endoscopic ultrasound procedure, including fine-needle aspiration, in the same session as ERCP; (6) contraindications for endoscopy, including severe cardiopulmonary disease; and (7) pregnancy.

#### 2.1.2. Sample Size Calculation

A previous study [7] reported that the success rate of biliary cannulation using a 0.025-inch CGW was approximately 80%. Considering a difference in the clinically significant success rate of 15% or less, with an alpha error of 0.05 and beta error of 0.20, 87 patients per group were required. Taking into account a dropout rate of 10%, a total of 192 patients were required.

#### 2.1.3. Study Outcomes and Definitions

The primary outcome was the selective biliary cannulation rate in naïve papillae. Biliary cannulation was defined as the insertion of the guidewire into the common bile duct. If the endoscopist failed biliary cannulation within 5 min, contacted the ampulla five times, or inserted three or more pancreatic duct cannulations, this was judged as a primary failure, and a rescue method was performed for selective cannulation.

The secondary outcomes were to measure the basic properties of the NGW and compare them with those of the CGW to analyze the importance of basic property differences. In addition, we also evaluated the biliary cannulation time, ERCP-related adverse events, and factors associated with successful primary biliary cannulation. The Cotton criteria were used to assess ERCP-related adverse events and their seriousness [12]. The shape of the ampulla was defined as described previously [13].

#### 2.1.4. Procedure

The ERCP was performed using a duodenoscope (TJF or JF 260, Olympus Medical, Tokyo, Japan; ED-580XT, Fuji Film, Tokyo, Japan). The tip shape (curved or straight) was determined according to the endoscopist’s preference. A cannula catheter (MTW Enoscope, Wesel, Germany) or CleverCut3V (Olympus Medical, Japan) was used. Biliary cannulation was performed using a guide wire-assisted technique. Moreover, the guidewires were manipulated by an assistant nurse who had expertise. If primary cannulation failed, a rescue method such as the double guidewire method, needle knife fistulotomy, and septostomy, was selected based on the endoscopist’s preference [14,15]. We evaluated the selective biliary success rate, attempted cannulation, frequency of pancreatic duct insertion, rescue methods, and adverse events, including PEP.

### 2.2. Basic Study

#### Basic Property Testing

The upper plateau stress (UPS) was measured at 3% strain during tensile loading of the Nitinol wire, according to the method described in ASTME F2516. The lower plateau stress (LPS) was measured at 2.5% strain during tensile unloading of the Nitinol wire, after loading to 6% strain per the method described in ASTME F2516 (Figure S1). For friction measurement, after putting the guidewire in a water bath at 37 °C, the friction load was measured by pulling the guidewire at a speed of 0.2 cm/s while applying a constant load on the wire with a silicon pad clamp at 50 gf force (Figure S2). Tip stiffness was measured at several length points at the distal tip part using a push–pull gauge (Figure S3).

### 2.3. Statistical Analysis

Statistical analysis was performed using IBM SPSS Statistics (version 21.0, IBM Corp., Armonk, NY, USA). Categorical data were expressed as frequencies and percentages, and between-group differences were evaluated using the chi-square test. Continuous data were expressed as the mean ± standard deviation, and between-group differences were evaluated using an independent Student’s *t*-test. Statistical significance was set at *p* < 0.05. Univariate and multivariate analyses were conducted to identify the factors associated with successful biliary cannulation. Variables with a *p* < 0.150 in the univariate analysis were included in the multivariate analysis.

## 3. Results

### 3.1. Basic Property Testing

The results of the basic technical tests are presented in Table 1. The two guidewires had the same length, sheath coating, and core-wire materials. The NGW had a greater tip length and smaller tip diameter than the CGW (80 mm vs. 50 mm and 0.57 mm vs. 0.59 mm, respectively) and showed better elastic resiliency, with larger UPS and LPS values. Theoretically, the larger the UPS and LPS, the better the elastic resilience. Furthermore, compared with the conventional Jagwire, the TargetSure guidewire had a higher maximum friction (34.6 ± 1.34 vs. 30.2 ± 4.09) and lower tip stiffness.

### 3.2. Baseline Characteristics

**Figure 2:**
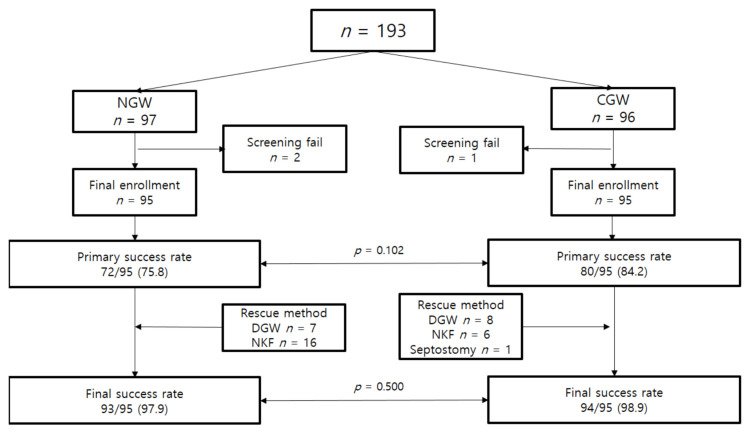
Flow chart of the study. NGW, newly developed guidewire; CGW, conventional guidewire; DGW, double guidewire technique; NKF, needle knife fistulotomy.

A total of 193 patients were screened, three of whom failed screening due to duodenal stricture. Ultimately, 190 patients were enrolled in the study, with 95 patients in each group. The study flowchart is shown in Figure 2.

There were no significant differences in the baseline characteristics between the two groups (Table 2). Of the laboratory parameters evaluated, only serum alkaline phosphatase and gamma-glutamyl transferase levels were significantly higher in the CGW group (*p* = 0.050 and *p* = 0.026, respectively; Table S1).

### 3.3. Outcomes

The study outcomes for each guidewire are shown in Table 3. The primary biliary cannulation success rates were similar in both groups (75.8% vs. 84.2%, *p* = 0.102), and the final cannulation success rates after rescue were also not significantly different (97.9% vs. 98.9%, *p* = 0.500).

Compared with the CGW group, the NGW group had a significantly higher frequency of ampulla contact (2.58 vs. 2.02, *p* = 0.011) and longer cannulation time (216.5 vs. 135.1 s, *p* = 0.016). There were no significant differences in the remaining procedure-related secondary outcomes. The adverse event rates, including PEP (6.3% vs. 4.2%, *p* = 0.374), hyperamylasemia (5.3% vs. 4.2%, *p* = 0.500), and cholecystitis (2.1% vs. 1.1%, *p* = 0.500), were also similar between the groups. All adverse events were mild and resolved with supportive care. No bleeding or perforation was observed in any of the patients.

Table 4 shows the factors associated with successful primary biliary cannulation. A total of 152 patients underwent successful primary biliary cannulation. In the univariate analysis, malignancy, periampullary diverticulum, normal papilla, small papilla, curved guidewire, and NGW were significant factors. In the multivariate analysis, normal papilla (odds ratio [OR], 0.39; confidence interval [CI], 0.17–0.86; *p* = 0.021) and curved tip (OR, 0.26; CI, 0.11–0.62; *p* = 0.002) were identified as factors that significantly influenced the success of primary biliary cannulation.

### 3.4. Success Rate According to Guidewire Tip Type

**Figure 3:**
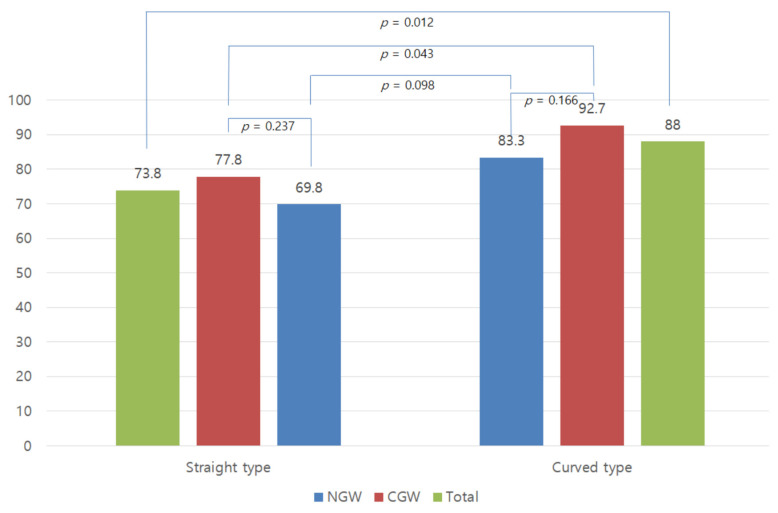
Primary success rate according to the type of guidewire.

In the logistic regression analysis, the curved-tip guidewire type was an important factor for primary biliary cannulation success. Therefore, we evaluated these parameters further, as shown in Figure 3. The curved-tip type had a significantly higher success rate than the straight-tip type guidewire (88% vs. 73.8%, *p* = 0.012). In the subgroup analysis, the curved-tip type also had a higher success rate in the CGW group (92.7% vs. 77.8%, *p* = 0.043). In the NGW group, there was no significant difference; however, success rates tended to be higher with the curved-tip type guidewire (83.2% vs. 69.8%, *p* = 0.098). For the same types of NGW and CGW, the success rates were similar (CGW vs. NGW, 92.7% vs. 83.3%, *p* = 0.166, curved-tip; 77.8% vs. 69.8%, *p* = 0.237, straight-tip).

## 4. Discussion

Our results s similar primary biliary cannulation rates with both guidewires, as well as similar final success and adverse event rates. However, the frequency of ampulla contact and the cannulation time significantly differed between the two groups. Furthermore, curved-tip-type guidewires and normal ampulla shape were associated with successful primary biliary cannulation.

Nitinol is a common engineering material used in the medical industry. It exhibits excellent superelastic and shape-memory properties as well as biocompatibility and has been extensively used in guidewires in recent years. The guidewire shaft should be elastic to ensure the forward axial transmission of forces [9]. Guidewires for biliary cannulation require a combination of different physical properties. The use of a nitinol core wire helps achieve this goal, but the characteristics vary slightly owing to different processing methods. Thus, further research is needed to determine which characteristics are most suitable. In the present study, compared with the CGW, the NGW showed higher friction and lower stiffness; therefore, it is considered that its cannulation ability is slightly lower, despite its high elasticity. As the guidewire must pass through a long catheter, low friction and high stiffness are required for easier cannulation. If these characteristics are supplemented while maintaining elasticity, a better guidewire for biliary cannulation is expected.

In the past, the contrast injection technique was the standard method for biliary cannulation. However, several studies have revealed that the guidewire-assisted technique has higher primary biliary success rates and lower adverse event rates, including post-ERCP pancreatitis, compared to the contrast injection technique [1,2,3]. At present, the guidewire-assisted technique is the standard method of biliary cannulation. Although a 0.035-inch guidewire was more frequently used because of its stiffness, recently, a high-performance 0.025-inch hydrophilic-coated guidewire has been developed and widely used [5,6]. In a recent meta-analysis of biliary cannulation according to the guidewire caliber [8], the primary biliary cannulation rates for the 0.035- and 0.025-inch guidewires were 82.0% and 80.6% (relative ratio, 1.02) and the PEP rates were 6.6% and 6.2% (relative ratio, 1.15), respectively. One study that was not included in the meta-analysis showed a similar biliary cannulation rate; however, the 0.025-inch guidewire had a lower frequency of ampulla contact and shorter cannulation time compared to those with the 0.035-inch guidewire [6]. The primary biliary cannulation rates (75.8% and 84.2%) and PEP rates (6.3% and 4.2%) in our study were similar to those reported in the above-mentioned studies.

In our study, normal ampulla shape and the curved-tip guidewire were associated with successful primary cannulation. The risk factors for difficult cannulation are well known, including the experience of the endoscopist [16], small and redundant ampulla [17], periampullary diverticulum [18], and surgically altered anatomy [19]. The structural abnormalities of the ampulla account for most of the risk factors. In a prior study, the normal ampulla had a shorter cannulation time and lesser frequency of ampulla contact than the variant ampulla, as well as a lower difficult cannulation rate (25% vs. 32.8–66.7%, *p* = 0.003) [20]. Hence, our result that the ampulla shape affected the cannulation success rate seems reasonable.

Few studies have compared biliary cannulation according to the guidewire tip type [10,11]. Three randomized controlled studies that were included in the meta-analyses used straight-tip guidewires [7,21,22]. One study compared curved- and straight-tip-type 0.035-inch guidewires and found no significant difference in the primary cannulation rate (curved vs. straight, 60% vs. 65%, *p* = 0.61); however, the success of primary cannulation was judged based on entry into the bile duct within 2 min [23]. In our study and in other studies, the mean cannulation time was 120–200 s [6]. Therefore, that may not be a proper evaluation because the criteria for success within 2 min are too severe. In the present study, the curved-tip guidewire showed a higher biliary cannulation rate than the straight-tip guidewire, and this result showed a similar trend regardless of the guidewire type (NGW or CGW). One study using the curved-tip type showed a tendency toward a higher cannulation rate with the 0.025-inch guidewire (0.035-inch vs. 0.025-inch: 86% vs. 96%, respectively), while other studies using the straight-tip type reported higher cannulation rates with the 0.035-inch guidewire (0.035-inch vs. 0.025-inch: 82% vs. 80.6%, respectively) [6,8]. The ampulla has a papillary fold between the common bile and pancreatic ducts [24]. We believe that curved-tip guidewires are more suitable than the straight-tip type for this structure of the ampulla. Namely, the tip of the guidewire rotates when torque is applied to the guidewire. A 0.025-inch guidewire is expected to better transmit torque because it has the same rigidity as a 0.035-inch guidewire but lower friction than other devices and the ampulla. These characteristics are helpful for advancing guidewires through the papillary folds in the ampulla.

Our study has several limitations. First, it was a non-inferiority study, and it was difficult to draw conclusions because the number of patients was relatively small. There were no significant differences between the NGW and CGW; however, the NGW demonstrated a lower success rate than the CGW. Thus, further studies are required to confirm their performance. Second, we did not perform a preliminary study to calculate the appropriate sample size. Thus, our study has limited meaning in its results due to the improper sample size calculation. The biliary cannulation rate of the CGW is well known, so we only tried to determine that the new product was not inferior to this product. Third, the curved-tip guidewire had a significantly higher success rate for primary biliary cannulation; however, it was not controlled by randomization but selected according to the endoscopist’s preference. Each endoscopist selected the guidewire type. Nonetheless, all of the endoscopists were experts who had performed over 5000 ERCP procedures and were not involved in fellowships during the procedure. There was no difference in the expertise between the endoscopists.

In conclusion, the NGW demonstrated similar success and adverse event rates as the CGW but showed a higher frequency of ampulla contact and longer cannulation time. Furthermore, a curved guidewire tip and normal ampulla shape were associated with higher primary biliary cannulation rates. However, further studies are needed to confirm our findings.

## Figures and Tables

**Table 1:** Results of the specification and basic technical test for the guidewire.

	Target Sure	Jagwire Revolution
Length (cm)	450	450
Tip length (mm)	80	50
Tip diameter (mm)	0.57	0.59
Core wire material	Nitinol	Nitinol
Sheath coating material	PTFE *	PTFE
Tip inner coil material	Gold-coated tungsten coil	N/A
Core wire diameter (mm)	0.548	0.582
UPS (MPa) **	587	476
LPS (MPa) ***	365	306
Tensile strength (MPa) ^$^	1394	1415
Measured max. friction force (gf) ^$$^	34.6 ± 1.34	30.2 ± 4.09
Calculated friction coefficient ^$$$^	0.692 ± 0.027	0.604 ± 0.082
Tip stiffness according to the length point (gf) ^$^		
At 20 mm	9.00 ± 0.110	8.54 ± 0.078
At 30 mm	4.37 ± 0.015	4.79 ± 0.017
At 40 mm	2.52 ± 0.036	3.51 ± 0.040
At 50 mm	1.69 ± 0.009	3.16 ± 0.018

* PTFE: polytetrafluoroethylene. ** UPS (upper plateau stress). *** LPS (upper plateau stress). ^$^ The data are shown as the mean value ± standard deviation (*n* = 5). ^$$^ Friction foefficient: max. friction force (gf)/clamp force (gf). ^$$$^ The clamp force condition is 50 gf.

**Table 2:** Baseline characteristics.

	NGW (*n* = 95)	CGW (*n* = 95)	*p*-Value
Sex (male)	57 (60.0)	48 (50.5)	0.122
Age	67.3 ± 13.8	65.6 ± 15.4	0.417
Body weight (kg)	64.3 ± 13.1	63.3 ± 13.5	0.591
Height (cm)	162.5 ± 8.8	162.2 ± 9.8	0.802
Body mass index (kg/m^2^)	24.2 ± 3.6	23.9 ± 3.3	0.480
**Disease**			0.631
Stone/CBD ca/GB ca	52(54.7)/7(7.4)/5(5.3)/	58(61.1)/4(4.2)/2(2.1)/	
/P-Ca./others	5(5.3)/26(26.3)	5 (5.3)/26(27.4)	
**PEP risk factors**			
IPMN	3 (3.2)	0 (0)	0.123
Age under 35	3 (3.2)	4 (4.2)	0.500
Normal bile duct diameter	40 (42.1)	36 (37.9)	0.328
Diameter over 10 mm	36 (37.9)	36 (37.9)	0.604
Normal bilirubin level	41 (43.2)	34 (35.8)	0.187
Hx of pancreatitis	0 (0)	1 (1.1)	0.500
r/o SOD	1 (1.1)	4 (4.2)	0.184
PAD	29 (30.5)	40 (42.1)	0.166
PAD type I/II/III	4(4.2)/16(16.8)/9(9.5)	4(4.2)/23(24.2)/13(13.7)	0.394
Normal papilla shape	54 (56.8)	52 (54.7)	0.564
Papillitis	9 (9.5)	11 (11.6)	0.547
Bulging	13 (13.7)	13 (13.7)	0.605
Redundant	18 (18.9)	12 (12.6)	0.285
Small ampulla	8 (8.4)	12 (12.6)	0.396

NGW, newly developed guidewire; CGW conventional guidewire; CBD, common bile duct; ca, cancer; GB, gallbladder; P-ca., pancreatic cancer; IPMN, intraductal papillary mucinous neoplasm; Hx, history; SOD, sphincter of oddi dysfunction; PAD, peri-ampullary diverticulum; PEP, post-ERCP pancreatitis.

**Table 3:** Result of each guidewire in biliary cannulation.

	NGW (*n* = 95)	CGW (*n* = 95)	*p*-Value
**Factor Related to the Procedure**			
Ampulla contact	2.58 ± 1.67	2.02 ± 1.33	0.011 *
P-duct cannulation	14 (14.7)	14 (14.7)	0.605
Cannulation number	1.35 ± 0.63	1.28 ± 0.46	0.737
P-duct contrast	0 (0)	2 (2.1)	0.249
ERPD	11 (11.6)	11 (11.6)	0.589
Cannulation time (s)	216.5 ± 293.2	135.1 ± 144.6	0.016 *
Procedure time (s)	1024.3 ± 1049.1	848.4 ± 636.8	0.165
Guidewire type			0.500
Straight/curved	53(55.8)/42(44.2)	54(56.8)/41(43.2)	
**Result of the Procedure**			
Primary success rate	72 (75.8)	80 (84.2)	0.102
Alternation method DGW/NKF/septostomy	7/16/0	8/6/1	
Final success rate	93 (97.9)	94 (98.9)	0.500
**Adverse Events**			
PEP	6 (6.3)	4 (4.2)	0.374
Hyperamylasemia	5 (5.3)	4 (4.2)	0.500
Bleeding	0 (0)	0 (0)	N/A
Perforation	0 (0)	0 (0)	N/A
Cholecystitis	2 (2.1)	1 (1.1)	0.500

NGW, newly developed guidewire; CGW conventional guidewire; p-duct, pancreatic duct; ERPD, endoscopic retrograde pancreatic drainage; DGW, double guidewire technique; NKF, needle knife fistulotomy; PEP, post-ERCP pancreatitis; *, *p*-value < 0.05.

**Table 4:** Multivariable analysis associated with the success of primary biliary cannulation.

	Total	Primary Success	Failed	*p*-Value		Odds Ratio
	*n* = 190	*n* = 152	*n* = 38	Univariate	Multivariate	
Sex (male)	105 (55.3)	87 (57.2)	18 (47.4)	0.181		
Age	66.4 ± 14.6	65.9 ± 14.6	68.3 ± 14.6	0.377		
Body mass index	24.0 ± 3.4	23.8 ± 3.1	24.6 ± 4.3	0.250		
Malignancy	28 (14.8)	19 (12.6)	9 (23.7)	0.076	0.709	1.41 (0.54–3.67)
IPMN	3 (1.6)	3 (2.0)	0 (0)	0.510		
Age under 35	7 (3.7)	6 (3.9)	1 (2.6)	0.575		
Normal bile duct diameter	76 (40.0)	59 (38.8)	17 (44.7)	0.313		
Diameter over 10 mm	72 (37.9)	59 (38.8)	13 (34.2)	0.372		
Normal bilirubin level	75 (39.5)	59 (38.8)	16 (42.1)	0.424		
Hx of the pancreatitis	1 (0.5)	1 (0.7)	0 (0)	0.800		
r/o SOD	5 (2.6)	4 (2.6)	1 (2.6)	0.738		
PAD	69 (36.3)	59 (38.8)	10 (26.3)	0.105	0.678	1.20 (0.53–2.79)
Normal papilla	107 (56.3)	89 (58.6)	18 (47.4)	0.145	0.021	0.39 (0.17–0.86)
Papillitis	20 (10.5)	18 (11.8)	2 (5.3)	0.191		
Bulging	26 (13.7)	20 (13.2)	6 (15.8)	0.422		
Redundant	30 (15.8)	22 (14.5)	8 (21.1)	0.223		
Small papilla	20 (10.5)	13 (8.6)	7 (18.4)	0.075	0.179	2.17 (0.70–6.71)
Guidewire type Curved	83 (43.7)	73 (48.0)	10 (26.3)	0.012	0.002	0.26 (0.11–0.62)
Group NGW	95 (50.0)	72 (47.4)	23 (60.5)	0.102	0.101	1.88 (0.88–3.98)

IPMN, intraductal papillary mucinous neoplasm; SOD, sphincter of oddi dysfunction; PAD, peri-ampullary diverticulum; NGW, newly developed guidewire.

## Data Availability

All relevant data contained within the article and Supplementary Materials.

## Supplementary Materials

The following supporting information can be downloaded at: https://www.mdpi.com/article/10.3390/jcm12103440/s1. Table S1: Laboratory finding for each group, Figure S1: upper and lower plateau stress test, Figure S2: Guidewire friction test, Figure S3: Tip stiffness according to the length point of guidewire distal tip.
