# Supplementary material for: Efficacy of a Newly Developed Guidewire for Selective Biliary Cannulation: A Multicenter Randomized Controlled Trial
Source: J Clin Med. 2023 May 12;12(10):3440. doi: 10.3390/jcm12103440 (PMC10218990; doi:10.3390/jcm12103440)

Supplementary Materials

Supplementary Table S1. Laboratory finding for each group.

	NGW (n=95)	CGW (n=95)	p-value
WBC	7.5 ± 3.5	8.4 ± 4.2	0.112
Hemoglobin	12.5 ± 1.9	12.5 ± 2.0	0.783
Platelet	228.0 ± 76.6	237.1 ± 81.8	0.429
INR	1.08 ± 0.19	1.08 ± 0.13	0.954
Total bilirubin	3.56 ± 4.88	3.97 ± 5.72	0.595
AST	233.2 ± 312.5	190.9 ± 243.5	0.300
ALT	219.5 ± 294.4	196.7 ± 214.8	0.541
ALP	229.5 ± 185.9	298.8 ± 284.5	0.050
rGT	370.5 ± 358.3	524.4 ± 539.2	0.026
Amylase	63.0 ± 33.5	68.4 ± 48.1	0.404
Lipase	58.3 ± 44.6	65.3 ± 61.9	0.409

Supplementary Figure S1. Upper and lower plateau stress test.

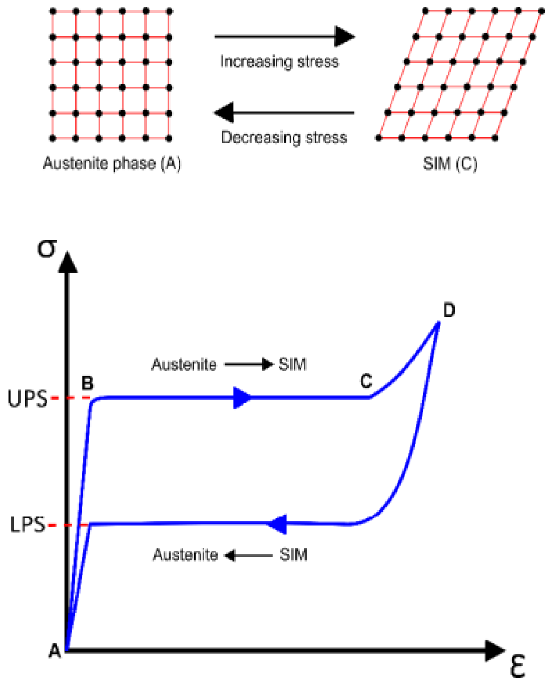

Supplementary Figure S2. Guidewire friction test.

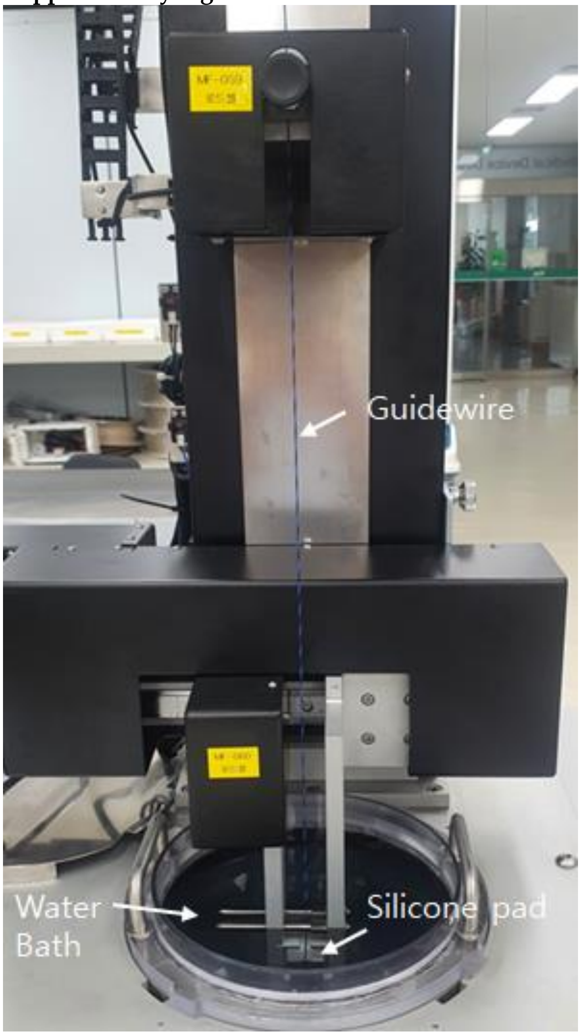

Supplementary Figure S3. Tip stiffness according to the length point of guidewire distal tip.

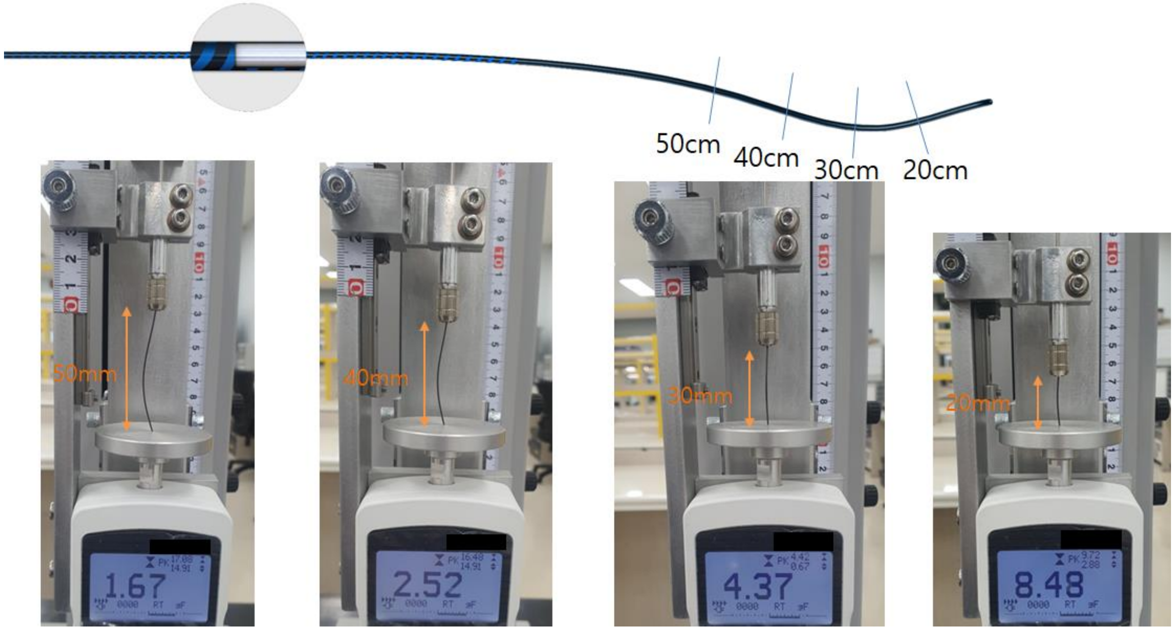

Supplement: Supplementary file 1 [file jcm-12-03440-s001.zip › jcm-2365330-supplementary.pdf]
